# Vaccination hesitancy: fear, trust, and exposure expectancy of an Ebola outbreak

**DOI:** 10.1016/j.heliyon.2019.e02016

**Published:** 2019-07-18

**Authors:** Gustavo S. Mesch, Kent P. Schwirian

**Affiliations:** a University of Haifa, Israel; b The Ohio State University, USA

**Keywords:** Fear, Trust, Expectancy, Vaccination hesitancy, Exposure expectancy scale, Psychology, Sociology, Public health

## Abstract

The purpose of this paper is to examine the relationship between vaccination hesitancy and fear, trust, and expectation of a potential imminent and proximate outbreak of Ebola. Our hypothesis is that people engage in self-protective behavior against an infectious disease when they are: fearful about things in general; trustful of government's ability to control the disease outbreak; and anticipating a direct threat to their health. The self-protective behavior we examine is the intention to accept a prospective anti-Ebola vaccination. We examine these relationships with basic demographic variables taken into account: gender, age, ethnicity, race and education. The data source is a national random sample of 1,018 United States adults interviewed early during the 2014 Ebola outbreak. We constructed a new three-item Exposure Expectancy Scale (alpha = 0. 635) to measure the degree of respondents' expectancy of a potential nearby Ebola outbreak. Our data analysis employs multiple logistic regressions. The findings support our hypothesis: willingness to take the Ebola vaccination is positively associated with a generalized sense of fear, trust in the government's ability to control an outbreak of the disease, and expectation of a potential Ebola outbreak that is imminent and proximate. The addition of the exposure expectancy variable in this analysis adds significantly to our understanding of contributors to vaccine hesitancy.

## 1 Introduction

Vaccination has become a principal public health response to the growing number of contagious diseases that infect the worlds’ population. Hesitancy refers to the delay in acceptance or refusal of accepting vaccine despite the availability of vaccination services (MacDonald and SAGE Working Group on Vaccination Hesitancy, 2015). Vaccine hesitancy is a complex phenomenon. It varies by several factors: disease, vaccine, time, place, and cultural context; furthermore, many demographic and behavioral factors influence vaccine hesitancy. The Sage Working Group has suggested that there are three major domains of variables that influence vaccine hesitancy; they are *convenience* or the ease of access, *confidence* or trust in the safety or efficacy of the vaccine, and *complacency* or risk of disease and importance of immunization (Larson et al., 2015).

The long history of vaccination has been characterized by hesitancy or refusal to be vaccinated in many countries and in many cases of disease including polio, pertussis, measles, and tetanus (Wolf and Sharp, 2002). In the US the immunization of children for DPT has spawned a nascent antivaccination movement. Although pro-vaccine and vaccination groups have emerged to contend with the anti-vaccination movement they do not get as much media coverage (Vanderslott, 2019). R.E. Spier (2001) argues that this is so because successful prevention of disease events is more easily ignored by the media than is news of serious disease outbreaks. The conflict between pro and anti-vaccination movements is not limited to the US but has emerged in many other countries as well including the UK, Japan, Canada, France, Philippians, and Kenya (MacDonald, 2017). The World Health Organization has listed both the anti-vaccination movement and Ebola among the top ten threats to global health in 2019 (Aranda, 2019).

While many specific factors have been shown to have significant relationships to vaccine hesitancy (Dubé et al., 2013), in this paper we suggest that, in addition to the basic variables of fear and trust of government (Heymann, 2017; Hofman and Au, 2017) another important factor correlates with vaccine hesitancy that has not been explicit in prior studies. That factor is the degree of expectancy of a potential imminent and proximal Ebola outbreak. We included this variable in the analysis, and we developed a reliable empirical three-item scale to measure it. Our study focuses on the early stages of the Ebola outbreak of 2013–2016.

### 1.1 Context

The Ebola outbreak initially took place in December 2013 in southern Guinea, Liberia, Sierra Leone, and Nigeria. By 2015 the World Health Organization reported there were 11,314 deaths and 28,634 cases (World Health Organization, 2015). The World Health Organization Response team placed the early case fatality rate at 70%.

Even though the outbreak was heavily concentrated in West Africa, with only four cases and one death reported in the United States, the press coverage was very intense in the leading media sources (Basch et al., 2014; Kelly et al.*,* 2015). Later the media were heavily criticized as fanning the hysteria, frenzy, and fear over Ebola Virus Disease (EVD) (Innes, 2015). The spreading word about Ebola was not limited to the press; it had exploded via Twitter as well. Between September 16, 2014 and Oct 6, 2014, 10.5 million tweets mentioning Ebola were sent (Luckerson, 2014). The net effect of news coverage and Twitter was that Ebola became ranked by the American public as one of the country's top three “urgent” health concerns (Hickey, 2014). Also nationwide polls showed that 68 percent of the respondents were either very concerned or somewhat concerned about a large possible Ebola outbreak in the U.S. within the next year (Herrnson and Weldon, 2014). Ebola vaccine was unavailable to the public (WHO, 2015). Without a vaccine there was a general uncertainty as to the appropriate adaptive behavior to avoid infection (Kanapathipillai et al., 2014; Kelly et al., 2015). By contrast, the level of worry about possible infection from Ebola far exceeded the level of worry in the general population for infection from H1N1 five years earlier--44.8 percent (Mesch et al., 2013). We suggest that the public social images of Ebola and H1N1 were quite different from each other and that difference contributed to a difference in public fear of infection from them. From press coverage Ebola had a more lethal image than did H1N1 (Mondragon et al., 2016).

In sum, the context of this study was a situation in which an American population was: (1) largely aware of the lethal EVD outbreak in Africa; (2) aware that some cases had shown up in the U.S.; and (3) concerned that a large Ebola outbreak could follow in the absence of an imminent prospect of immunization.

### 1.2 Theoretical model and hypotheses

In addition to being informed by past studies in explaining vaccine hesitancy, we draw on two related theoretical models: Social Cognition Theory and Protection Motivation Theory. Social Cognition Theory (SCT) is rooted in the work of Albert Bandura (1986). The theory argues that in matters of health behavior there is reciprocal triadic causation among three basic components: behavior, person, and environment; that is, each of the three affects the other two (Crosby2013). Environment consists of all factors that act on individual behavior including social, economic policy, legal or physical matters. The environment, in turn, is shaped by both individual and collective agency which then feeds back on individual behavior. The environment may also influence an individual's perceptions and outcome expectations. The link between person and behavior indicates the fact that behavior is tied to a complex set of personal factors (age, race, status, etc.) whose mix and configuration shapes behavior. It is the uniqueness of this configuration between people that contributes to behavioral differences among them. Thus, the person-behavior link asserts that individual cognition shapes one's behavior. As Jazen et al. (2006) have summarized, “If the outcome expectancy is positive, the behavior required to attain it is more likely to be engaged in than if the outcome expectancy is negative.” Thus if people perceive that vaccination will have positive results, they will tend to strive to attain it.

From the standpoint of this study, SCT leads us to focus on how the perceived proximity of the environmental location of an Ebola outbreak impacts individuals' coping behavior over and above the influences of their individual modifying factors. That is, the model predicts that as the environment is changed by the locational intrusion of a disease outbreak, the individual's coping behavior responds and is changed. The coping behavior examined in this study is the decision to accept or not to accept the prospective Ebola vaccination.

The second theory on which we draw is Protection Motivation Theory (PMT) proposed by Ronald Rogers (1975, 1983). Over the years PMT has proven to be especially helpful in the study of fear and health-related behaviors (Milne et al., 2000). Basic PMT theory states that the individual's intent to adopt a response to a threatening situation is formed by: the perceived magnitude of the threat; the probability of the threat's occurrence; and the efficacy of the coping response (Rogers, 1975). As those factors increase, the motivation to engage in protective behavior increases and that, in turn, positively affects the intent to adopt recommended responses such as vaccination.

From the standpoint of this study, PMT directs our attention to the public health recommended response for an Ebola outbreak which is the willingness to accept a vaccination against Ebola when it becomes available. PMT suggests that this willingness is influenced by the degree of fear the respondent carries toward the disease and the perceived likelihood of its near-by outbreak. Willingness also is affected by the respondent's confidence in the efficacy of the government to successfully prevent a nationwide epidemic of Ebola.

Our general hypothesis is that people engage in protective adaptive behavior when an actual or impending threat to their health is perceived. More specifically:

H1 The more fearful people are, the more likely they are to commit to getting a vaccination for Ebola.

H2 The more trustful people are of the federal government's ability to prevent a national epidemic, the more likely they are to commit to vaccination for Ebola

H3 As the perceived likelihood of an imminent and proximate Ebola outbreak increases, the more likely people are to commit to getting a vaccination for Ebola.

## 2 Methods

### 2.1 Sample

We conducted a secondary data analysis of the CNN/ORC Poll: Terrorism/ISSIS/Ebola, October 2014 [dataset]. USORCCNN 2014-010, Version 2. Opinion Research Corporation [producer]. Storrs, CT: Roper Center for Public Opinion Research, Roper *Express* [distributor], accessed Feb-1-2016*.* This random sample relied on both landline telephones and cellular phones. The sample was nationwide and consisted of 1018 cases. Members of the sample were interviewed during the early period of the Ebola outbreak when they were just learning about the outbreak, October 24-October 26, 2014. No vaccine was yet available (Center for Disease Control and Prevention, 2018). The Poll received the informed consent from all participants in the study and no personal identifying information is included in the data file. There were no additional institutional ethical requirements for the authors.

### 2.2 Variables

There are three categories of variables: dependent variable, key independent variables, and modifying variables.

### 2.3 Dependent variable

Each member of the sample was asked:

“If a vaccine that protects people from the Ebola virus became available, would you yourself get the shot?” At this time the sample was divided on their willingness to take the vaccine: Yes (48.2%), No (48.7%). Willingness to take the shot was considered an adaptive behavior.

### 2.4 Independent variables

There are three independent variables: fear, trust, and the proximity expectancy of an outbreak of Ebola. To measure fear each respondent was asked:

“And would you say you are very scared (27.0%), somewhat scared (34.9%), not very scared (18.7%) or nor not scared at all (19.0%) about the way things are going in the country today?” Over 60 percent of respondents were either very or somewhat scared. Table 1 shows that as fear increases, the percentage willing to take the Ebola vaccine increases (p = .001).

**Table 1:** Percentage willing to take vaccine by variable.

Variable	Total N in Table (Percent)	Percentage Willing to Take Vaccine (Yes = 48.2%) (No = 48.7%)
*Gender*		*p = .*940
Male	511 (50.2)	50.71
Female	507 (49.8)	49.29
*Age*		*P =* .002
18–29	119 (11.9)	12.02
30–49	220 (22.0)	21.18
50–64	324 (32.3)	29.94
65+	339 (33.8)	35.64
*Race and Ethnicity*		*P=*.141
White	761 (74.8)	47.96
African American	90 (8.8)	54.44
Hispanic	54 (5.3)	55.56
Other	115 (9.0)	41.59
*Education Level*		*P* = .021
Less than High School	52 (5.1)	63.46
High School Grad	295 (29.0)	54.24
Some College	259 (25.3)	45.74
College Grad & Beyond	401 (39.4)	42.89
*Confidence Federal Government Prevent Outbreak*		*P* = .359
Very Confident	328 (32.2)	46.34
Somewhat Confident	414 (40.6)	50.48
Not Too Confident	170 (16.7)	50.59
Not at All Confident	103 (10.1)	40.78
*Scared about How Things are Going in the U.S.*		*P =* .001
Very Scared	275 (27.0)	53.09
Somewhat Scared	355 (34.9)	50.99
Not Very Scared	190 (18.7)	43.68
Not Scared at All	193 (19.0)	41.97
*Likely a Person in the U.S. Will be Infected*		*P =*.624
Very Likely	471 (46.3)	50.96
Somewhat Likely	351 (34.5)	47.58
Not Too Likely	136 (13.4)	43.38
Not at All Likely	58 (5.7)	43.10
*Likely a Person in your Local Community Will be Infected*		*P=* .001
Very Likely	55 (5.4)	65.45
Somewhat Likely	180 (17.7)	51.67
Not Too Likely	370 (36.4)	52.70
Not at All Likely	408 (40.1)	40.69
*Likely a Person in the your immediate family Will be Infected*		*P =* .001
Very Likely	8 (0.8)	75.00
Somewhat Likely	49 (4.8)	61.22
Not Too Likely	255 (25.0)	52.94
Not at All Likely	700 (68.8)	45.43

The second independent variable is confidence in the federal government's ability to prevent an Ebola epidemic. Each respondent was asked: “How confident are you that the federal government can prevent a nationwide epidemic of the Ebola virus—very confident (32.2%), somewhat confident (40.6%), not very confident (16.7), not confident at all (10.1%)”. Thus over 70 percent of the sample members were confident in the government's ability to prevent a massive outbreak of Ebola. Table 1 shows that those respondents expressing the lowest level of confidence were less likely to take the Ebola vaccine than were others.

Interestingly, confidence in the government's ability to control an Ebola outbreak should one occur was related to the extent to which people expressed a generalized fear for things in society. The relationship was inverse. The probability was 0.001. Forty-one percent of those highly scared were very or somewhat confident in the government's ability while 31.8% of those also less fearful were confident in the government's ability. We suggest that while that majority of people were confident in the government given the positive nature of those responses in the face of virus outbreaks in the preceding ten years, however enough on the international and domestic economic and political scenes still lead the majority of people to express generalized fear. Trust was specific to medical action during outbreaks while being scared was a resultant of broad social and environmental conditions beyond the potential invasion of a remote viral disease.

The third independent variable is exposure expectancy. It is measured by a factor that combines three questionnaire items into a scale.

The first of the three items is: “How likely do you think it is that someone in the United States will be infected with the Ebola virus in the next few weeks—very likely (46.3%), somewhat likely (34.5%), not too likely (13.4%), or not likely at all (5.7%)”. Over 80% of the sample expected an Ebola outbreak somewhere in the United States in the near future.

The second question moved the likelihood of an outbreak closer to the respondent. It asked “How likely do you think it is that someone in your local community will be infected with the Ebola virus in the next few weeks—very likely (5.4%), somewhat likely (17.7 %), not too likely (36.4%), or not likely at all (40.1%)” While over three-fourths of the sample expected an Ebola outbreak somewhere in the U.S., a much smaller group expected an outbreak in their local community.

The third question moved the location of a potential outbreak even closer. The respondents were asked, “How likely do you think it is that you yourself or someone in your immediate family will be infected with the Ebola virus in the next few weeks—very likely (0.8%), somewhat likely (4.8%), not too likely (25.0%), not at all likely (68.8%). Thus, most of the sample expected an outbreak somewhere in the U.S. but not likely in their own community or family.

For each of the locations, as the likelihood of an outbreak increases so too does the willingness to take the vaccine. The three locations are consistent with Diggory (1956) classic spatial hypothesis that the more proximate of a disease threat, the greater the likelihood that people will engage in adaptive coping behavior.

We combined the three “proximity” questions into a scale that measures the respondents’ overall expectancy of an Ebola outbreak in the next few weeks. We conducted a factor analysis on the three “proximity” questions. Each of the three items had a significant loading on the extracted factor: “outbreak in U.S.” (loading = .516); “outbreak in the nearby city” (loading = .628); and “outbreak in family” (loading = .582). The overall scale reliability (Cronbach alpha) was 0.635. The loadings were used to calculate a scale score for each respondent. The scores ranged from 3 (high expected proximity) to 12 (low expectance). The mean expectancy was 8.54 with a standard deviation of 1.83. As the Exposure Expectancy increased, so too did the percentage willing to take the vaccine. The percentage willing to take the vaccine for those of higher expectation (n = 267) was 56.2; for those of medium expectation (n = 411) it was 50.1; for those of lower expectation (n = 301) it was 43.8. Those with the greatest expectation of an outbreak were the most likely to be willing to take the vaccination. .

### 2.5 Modifying variables

The modifying variables are: age, sex, education, race, and ethnicity. Table 1 shows that two of the modifying variables had a relationship with willingness to take the vaccine: age and education. The older respondents were more willing than were the younger to take the vaccine. We interpret this to reflect the fact that older persons in general are more aggressive in matters of a novel contagious disease such as an Ebola outbreak than are the younger. This age difference is consistent with findings of the 2018 seasonal influenza vaccination in the U.S (CDC, 2018).

Also, those with less formal education were more willing to take the vaccine than those of higher education. We contend that this suggests that individuals with more education are more conservative in selection of treatment protocols until more is known about Ebola. This inverse relationship between education level and willingness to take the vaccine has also been reported in other studies. For example (Mesch and Schwirian, 2015) report in a study of willingness to take the vaccine for H1N1, 43.3% of those with less than a high school level of education were willing to take the vaccine while 37.8% of those with some college or more were willing. Furthermore, others argue that the effect of education is not direct, but is mediated by perceived risk (Mesch and Schwirian, 2014).

### 2.6 Findings: multiple logistic regressions

Table 2 presents the multiple logistic regressions in three panels. Panel 1 shows the multiple regression of taking the Ebola vaccination on the three independent variables: Fear, Trust, and Expectancy. Each of the three has a significant relationship with taking the vaccination with the other two controlled.

**Table 2:** Logistic regressions of vaccine on independent variables (panel 1), modifying variables (panel 2), and all variables (panel 3).

Panel 1				
Independent Variables	Odds Ratio	Standard Error	Probability	95% Confidence
Fear	.855	.055	.015	.754–.970
Trust	.846	.064	.028	.730–.982
Exp.Expectancy	.858	.034	.001	.794–.924
N = 975	P = .0001	Pseudo R2 = .019	Log likelihood =	-663.057
Panel 2				
Modifying Variables				
Education	.664	.074	.001	.533–827
Age	1.025	.061	.682	.917–1.152
Sex	.961	.125	.758	.744–1.234
White	.965	.048	.985	.874–1.065
Af. American	.997	.172	.700	.711–1.397
Hispanic	.933	.168		.656–1.327
N = 975	p = . 0101	Pseudo R2 = .012	Log likelihood =	-667.386
Panel 3				
All Variables				
Fear	.870	.059	.038	.762–.992
Trust	.826	.064	.013	.710–.961
Exp. Expectancy	.853	.034	.001	.543–.853
Education	.680	.079	.001	.885–1.127
Sex	.911	.121	.482	.702–1.182
White	.976	.051	.640	.881–1.081
Af. American	1.051	.193	.666	.761–1.532
Hispanic	.856	.159	.402	.595–1.232
N = 963	P = 0001	Pseudo R2 = .030	Log likelihood =	-647. 389

Panel 2 shows the results from the multiple regression of willingness to take the Ebola vaccination on the six modifying variables: Education, Age, Sex, White, African American, and Hispanic. Age is no longer significant with the other modifying variables in the equation, but the effect of Education continues to remain significant.

Panel 3 shows the multiple regression of willingness to take the Ebola vaccination on all the variables-both independent and modifying. Each independent variable has a significant relationship with vaccination willingness with all of the modifying variables controlled; Fear (AOR = .870, CI95 = .762-.992, p < .038)), Trust (AOR = .826, CI95 = .710-.961, p < .013), and Exposure Expectancy (AOR = .835, CI95 .543-.853 = p < .001). The only control variable approaching significance is Education. The probability of the effect of Education is .001. However, the upper limit of Education's 95% confidence interval is 1.127. Normally, when the upper level of the AOR confidence exceeds 1.00 and the lower boundary is less than 1.00, the effect is considered non-significant and thus compatible with the null hypothesis.

## 3 Discussion

Each of this study's three hypotheses is supported by the data; that is willingness to take a prospective Ebola vaccine is related to a generalized sense of fear, to trust in the federal government's ability to prevent an outbreak of a little known killer disease, and to the extent to which an Ebola outbreak is anticipated to occur in the local environment. We suggest that the elements of confidence, compliancy, and convenience resonate in the findings. We see confidence in the 73 % of the sample willing to trust the government's ability to deal with an Ebola outbreak. We see compliancy in the 76% of the sample who doubt that a resident of their community will become infected with Ebola. And we see convenience in the fact that 48% of the sample is willing to take the Ebola vaccination even though its future degree of convenience is undetermined.

This paper introduces two operational measures not normally found in studies of vaccine hesitancy. We suggest that they provide stronger measures than do typical routine measures (Mesch et al., 2013). One is the measure of fear. The measure used here is more general than most others used in such studies. Fear of being infected is usually treated more specifically in terms of a single disease such as fear of Swine Flu, SARS, or Measles. We suggest that a generalized fearful orientation toward the way things are going in one's current life environment becomes reflected in a specific motivation to engage in health protective behavior such as willingness to take a prospective vaccine. Specifically, respondents were asked if they were “scared” about “…the way things are going in the country today”. We contend that “scared” carries a stronger connotation of fearfulness than is commonly measured and therefore more likely to be motivational and reflected in engagement in health protective behavior.

Our second novel measure is that of exposure expectancy of disease outbreak. Rogers (1975) suggested that expectancy of disease exposure was an important cognitive mediating factor in the intent to adopt a protective motivation behavior. Thus, protective motivation becomes reflected in intent to adopt the public-health recommendation of vaccination. We have used a geographically wider measure of expectancy than is commonly reported; that is, a three item scale rather than a one-variable assessment. The use of a scaling approach in this study is consistent with a more general attempt in this literature to incorporate more sophisticated measurement indexes into the research (Larson et al., 2015).

This study focused on a highly publicized outbreak of a killer disease in one distant part of the world. At the time of the outbreak it was not known if Ebola would reach the U.S. population and what its consequences might be. Fear of disease in the United States was high at the time and may have influenced respondents’ willingness to be vaccinated—assuming that a vaccine existed. Would fear be as effective in an Ebola outbreak today? At this writing, Ebola is once again largely confined to Africa; this time there is much less coverage in the American media. Moreover, progress has been made in development of a preventive vaccine for the disease. We contend that a re-examination of the research question is in order. That is, do fear, trust, and exposure expectancy relate in the same manner in the US population today as vaccine is soon to become available as they did in 2014 when vaccine was not available? Would having vaccine on hand make a greater willingness of respondents to take the vaccine? If there were an outbreak of Ebola in the U.S., we hypothesize that a larger percentage of the sample would opt for the vaccine.

Finally there is the matter of the social representation of Ebola. Nahia Mondragon et al. (2016) drawing on Social Representations Theory (SRT) argue that it is important to investigate how people incorporate the image of disease in general and Ebola in particular into their understanding of daily life events. This understanding influences their general fear response to the contagious outbreak, to the trust they hold in responding institutions, and to the behavioral coping mechanism they employ. In Mondragon's study of the collective social representation of Ebola that developed in Spain during 2014–2015 emphasized: the original nature of Ebola as an African disease that spread to become a frightening global health threat, a public health failure of the Spanish government by its ineptitude and corruption, general anger at the mass media for sensationalizing risk messages, and anger at pharmaceutical organizations for taking advantage of people's plight. Adding SRT to research may move a study further in explanatory analysis and provide a significant way to include the public's views in the development of an anti-Ebola program. To the extent that the public's views are include in the structure and delivery of the program, the greater the trust in the program, and the more likely the success of the program (Paterson and Larson, 2012).

## Declarations

### Author contribution statement

Gustavo S. Mesch, Kent P. Schwirian: Conceived and designed the experiments; Analyzed and interpreted the data; Wrote the paper.

### Funding statement

This research did not receive any specific grant from funding agencies in the public, commercial, or not-for-profit sectors.

### Competing interest statement

The authors declare no conflict of interest.

### Additional information

No additional information is available for this paper.
